# Landscape of the lipidome and transcriptome under heat stress in *Arabidopsis thaliana*

**DOI:** 10.1038/srep10533

**Published:** 2015-05-27

**Authors:** Yasuhiro Higashi, Yozo Okazaki, Fumiyoshi Myouga, Kazuo Shinozaki, Kazuki Saito

**Affiliations:** 1 RIKEN Center for Sustainable Resource Science, 1-7-22 Suehiro-cho, Tsurumi-ku, Yokohama, Kanagawa 230-0045, Japan; 2 Graduate School of Pharmaceutical Sciences, Chiba University, Inohana 1-8-1, Chuo-ku, Chiba 260-8675, Japan

## Abstract

Environmental stress causes membrane damage in plants. Lipid studies are required to understand the adaptation of plants to climate change. Here, LC-MS-based lipidomic and microarray transcriptome analyses were carried out to elucidate the effect of short-term heat stress on the *Arabidopsis thaliana* leaf membrane. Vegetative plants were subjected to high temperatures for one day, and then grown under normal conditions. Sixty-six detected glycerolipid species were classified according to patterns of compositional change by Spearman’s correlation coefficient. Triacylglycerols, 36:4- and 36:5-monogalactosyldiacylglycerol, 34:2- and 36:2-digalactosyldiacylglycerol, 34:1-, 36:1- and 36:6-phosphatidylcholine, and 34:1-phosphatidylethanolamine increased by the stress and immediately decreased during recovery. The relative amount of one triacylglycerol species (54:9) containing α-linolenic acid (18:3) increased under heat stress. These results suggest that heat stress in Arabidopsis leaves induces an increase in triacylglycerol levels, which functions as an intermediate of lipid turnover, and results in a decrease in membrane polyunsaturated fatty acids. Microarray data revealed candidate genes responsible for the observed metabolic changes.

Due to climate change, land plants may suffer heat stress, which causes an increase in molecular disorder and disintegration of lipid bilayers^1^,^2^. Heat stress-induced reactive oxygen species promote peroxidation of unsaturated fatty acids^3^. Plastidic fatty acid desaturase (*FAD*) mutants, in which the number of glycerolipid fatty acid double bonds is reduced, are known to be heat tolerant^4^,^5^,^6^. Another mutation resulting in impaired biosynthesis of digalactosyldiacylglycerol (DGDG) in chloroplasts has been shown to lead to heat sensitivity^7^. These mutant studies suggest that changes in the proportion of glycerolipid composition in chloroplasts are required for plant acclimatization to heat stress.

One study on Arabidopsis plants under various high temperatures suggested that the heat-induced decrease in unsaturation of membrane glycerolipids is slow and consistent with the rate of *de novo* fatty acid synthesis^8^. Therefore, a decrease in glycerolipid unsaturation is considered unsuitable for a rapid response (increase) to environmental temperature, but is advantageous for plants for long-term acclimatization to gradual temperature increases. A time-course study on Arabidopsis plants grown at 33 °C showed that α-linolenic acid (18:3) decreased within 3 days, whereas hexadecatrienoic (roughanic) acid (16:3) gradually decreased after an additional 13 days^6^. Metabolic labelling experiments have shown that incorporation of 1-[^14^C]-acetate into DGDG was slower than that into monogalactosyldiacylglycerol (MGDG) and phosphatidylcholine (PC)^9^. Because the rate of biosynthesis and degree of unsaturation of glycerolipids are different among lipid classes, it is useful to compare compositions of various lipid species under different temperatures and correlate metabolite patterns with metabolic pathways.

Plant leaves synthesise small amounts of triacylglycerol (TAG) in the endoplasmic reticulum (ER) and accumulate it as lipid droplets in the cytosol^10^. TAG is also possibly synthesised in chloroplasts and accumulated as plastoglobules in chloroplasts^11^. The accumulation of plastoglobules was observed after a short-term moderate heat treatment in Arabidopsis^12^, suggesting an increase in the accumulation of TAG under heat stress. Recent LC-MS-based lipidomic studies revealed that long-term cold stress and a 2-week drought stress resulted in increases in TAG species in Arabidopsis^13^,^14^. According to these data, the composition of the detected TAG species seems to be different among the stresses even in the same ecotype plants. During recovery from drought stress in *Craterostigma plantagineum*, the extent of the decrease differed among individual stress-induced TAG species^14^. These results suggest that each TAG species behaves differently during the environmental stress, possibly reflecting the change (decrease) in membrane glycerolipid composition.

The utility of the integration of metabolic changes with transcriptome data in gene discovery has often been reported in studies on plant specialized (secondary) metabolism^15^. Published microarray transcriptome data suggest the coexpression of genes involved in *de novo* fatty acid synthesis^16^, glycolipid synthesis^17^, and the β-oxidation/glyoxylate cycle^18^. Senescent leaves coordinately reduce and induce the expression of genes involved in *de novo* fatty acid synthesis and β-oxidation, respectively^19^. An integrated lipidomic and transcriptomic study in Arabidopsis under environmental stress was recently conducted^20^. Their results suggest that Arabidopsis transcriptionally coordinates the regulation of glycerolipid metabolism, and the metabolic changes under heat stress may be explained by induced and reduced expression of genes encoding enzymes, proteins involved in lipid trafficking, and transcription factors.

Plant membrane properties should be determined and explained as resulting from the complex mixture of lipid species having different headgroup structures, fatty acid lengths, and the degree of unsaturation. In this study, we simultaneously analysed the changes in glycerolipids including glycolipids, phospholipids, and storage lipids in Arabidopsis leaves under heat stress and during recovery by an LC-MS-based lipidomics platform. Although plant leaf lipidomic studies under heat stress have been reported^7^,^21^,^22^, a thorough determination of TAG species, changes during recovery, and integration with gene expression are required for a better understanding of heat responses in lipid remodelling. In the present study, lipid species were classified according to the pattern of compositional changes, and these changes were represented on a metabolic pathway map with possible lipid trafficking among cellular organelles. A microarray transcriptome analysis was performed to determine the molecular regulation of lipid metabolism under heat stress. We describe plant acclimatization responses to heat stress based on the changes in glycerolipid properties, and suggest an important role for TAG formation.

## Results and Discussion

### Lipidomics reveals a diversity of lipids accumulated under heat stress

Arabidopsis ecotypes Col-0 and Nossen were grown on agar plates and treated with one day of heat stress at 14 days of age (Supplementary Fig. S1). Four temperatures, 22 °C (control), 30 °C, 34 °C and 38 °C were examined. Nossen plants grown at 22 °C had a greater fresh weight than Col-0 plants grown at 22 °C. As compared to the plants grown at 22 °C, both ecotype plants at 30 °C and 34 °C showed more petiole length (Supplementary Fig. S2). Indeed, hyponastic growth is known to be positively regulated by the plant hormones auxin and abscisic acid under heat stress^23^,^24^. The 38 °C treatment was not lethal, but no considerable elongation was observed (Supplementary Fig. S2). Aerial parts were harvested and analysed using a lipidomics method. Lipid composition during recovery from heat stress was also examined. Col-0 plants at 18 days and Nossen plants at 14 days were subjected to 38 °C heat stress for one day. Aerial parts were harvested at one and two days after the stress treatment (Supplementary Fig. S1).

**Figure 1:**
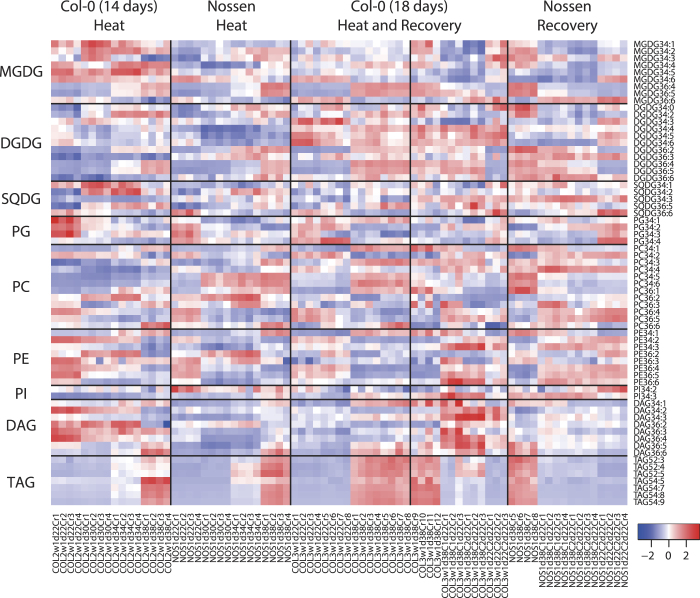
Overview of glycerolipid levels in Arabidopsis leaves under heat stress and during recovery. The 66 lipid species observed from the all environmental conditions (heat and recovery) and ecotypes (Col-0 and Nossen) were shown. Values were normalised to 1d38C data in each experiment, and scaled among measurements as described in Methods. Each column represents an LC-MS measurement. Biological replicates N = 3, 4, 8, or 12. MGDG, monogalactosyldiacylglycerol; DGDG, digalactosyldiacylglycerol; SQDG, sulfoquinovosyldiacylglycerol; PG, phosphatidylglycerol; PC, phosphatidylcholine; PE, phosphatidylethanolamine; PI, phosphatidylinositol; DAG, diacylglycerol; TAG, triacylglycerol. Experimental condition can be found in Supplementary Fig. S1. The normalised data was available in Supplementary Table S1.

Lipidomic analysis detected a number of glycerolipid species including (1) the glycolipids MGDG, DGDG, and sulfoquinovosyldiacylglycerol (SQDG); (2) the phospholipids phosphatidylglycerol (PG), PC, phosphatidylethanolamine (PE), and phosphatidylinositol (PI); (3) diacylglycerol (DAG), a lipid intermediate; and (4) the storage lipid TAG (Fig. 1, Supplementary Table S1).

Mass spectrometry was used to determine the total number of carbon atoms and double bonds of two fatty acids within a glycerolipid species. Arabidopsis leaves mostly accumulate glycerolipids containing the fatty acids 34:1, 34:2, 34:3, 34:6, 36:1, 36:2, 36:4, 36:5, and 36:6, which are likely composed of two fatty acid combinations: palmitic acid (16:0)/ oleic acid (18:1), 16:0/ linoleic acid (18:2), 16:0/18:3, 16:3/18:3, 18:0/18:1, 18:1/18:1, 18:2/18:2, 18:2/18:3 and 18:3/18:3, respectively^9^,^25^,^26^. Seventy lipid species ranging in number from 34:0 to 34:6 and 36:0 to 36:6 were observed in at least one environmental condition (Supplementary Table S1). Among them, 59 lipid species were observed in all conditions (Fig. 1).

TAG is composed of 3 fatty acids. Twenty-eight TAG species were observed in at least one environmental condition. The major TAG species in Arabidopsis leaves were TAGs consisting of 52:x and 54:x, which contain 52 and 54 carbon atoms in the 3 fatty acids in total. TAGs consisting of 50:x and 56:x, which contain 50 and 56 carbon atoms, respectively, were also detected, but occupied less than 3% of the total amount of TAG species in Arabidopsis leaves. Seven TAG species were detected in all conditions (Fig. 1).

### Lipid species can be classified by their patterns of change under heat stress

To classify the detected lipid species according to trends in accumulation changes, Spearman’s correlation coefficients were calculated among 12 variables of conditions and the result is shown in matrix form (Supplementary Fig. S3). Hierarchical clustering analysis using the complete linkage method based on Euclidean distance classified the 66 lipid species (59 diacyl glycerolipids and 7 TAG) into 10 groups. This result indicated that there were 3 pairs having strong negative correlations, i.e. the lipid species belonging to clusters 1 and 10, clusters 2 and 6, and clusters 6 and 7. The groups exhibiting a negative correlation are possibly interconverted during the stress treatment.

**Figure 2:**
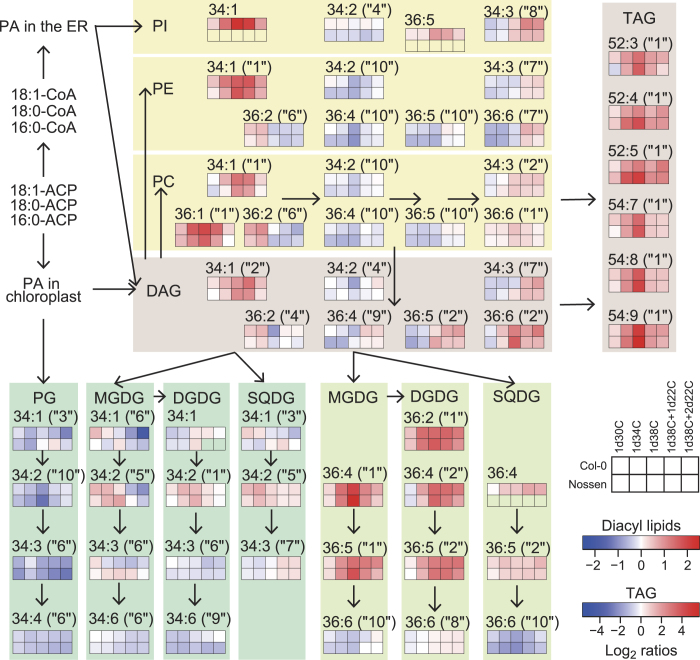
Heat map representation of lipid species in Arabidopsis leaves under heat stress superimposed on a glycerolipid metabolic pathway map. The average changes in lipid species are shown by 10 boxes (2 rows and 5 columns), which contain 2 genotypes: Col-0 (upper row) and Nossen (lower row); and 5 environments: 1d30C, 1d34C, 1d38C, 1d38C + 1d22C, 1d38C + 2d22C (left-to-right). Heat map colours show the average log_2_ ratios of fold-changes in each stress condition to the normal 22 °C condition. Due to different induction levels, values of diacyl membrane lipids and TAG are shown by different scales. Samples depicted by boxes with no colour in the Nossen data were not detected. Lipid species belonging to the 10 clusters (Spearman’s correlation coefficients, see Supplementary Fig. S3 for details) are shown with the numbers in parentheses (e.g. “1”, “2”, “6”, “7”, and “10”). Individual experimental data can be found in Supplementary Table S1.

The obtained metabolic changes were represented as a heat map superimposed on a metabolic pathway map (Fig. 2). Fifty-seven lipid species were selected based on their relatively high accumulation in Col-0 plants and their detectability in all conditions. Characteristic lipid species belonged to 5 clusters (clusters 1, 2, 6, 7 and 10). Cluster 1 represents the lipid species that were increased by heat stress and returned to normal levels during recovery. Cluster 2 represents the lipid species that were increased by heat stress and gradually returned to normal levels (more slowly than cluster 1) during recovery. Cluster 7 represents the lipid species that increased to above normal levels during recovery. Cluster 10 represents the lipid species that were decreased by heat stress and returned to normal levels during recovery. Cluster 6 represents the lipid species that decreased at 38 °C and further decreased or remained constant during recovery. Lipid species belonging to the same cluster tended to be closely situated in the metabolic pathway (Fig. 2). The reported clusters of Arabidopsis leaves after wounding^27^ were not reproduced well in our clusters after heat stress, suggesting the different response occurred under the different abiotic stresses in Arabidopsis leaves.

### TAG increased under heat stress and likely enhanced the changes in membrane composition

Although most of the detected TAG species increased under heat stress, the relative amounts of TAG species were also altered (Supplementary Fig. S4). The relative amount of 54:9 TAG increased along with increased temperature. The relative amount of 52:5 TAG peaked at 34 °C and decreased at 38 °C. During recovery, the relative amount of 52:5 TAG increased and that of 54:9 TAG decreased. Because 54:9 TAG is likely composed of three 18:3 chains, these data suggest that TAG synthesis under heat stress favours 18:3 as a substrate. Increase in the relative amount of 52:5 TAG has been reported in studies of Arabidopsis under long-term cold stress and during leaf senescence^13^,^28^. A slower decrease in 52:x TAGs during recovery from drought stress as compared to that of 54:x TAG in *C. plantagineum* has been reported^14^. Taken together, these results suggest that the increase in 54:9 TAG is more specific to short-term heat stress and the increase in 52:5 TAG represents a comprehensive and slow response to environmental stress.

It has been suggested that the ER glycerolipid pathway and TAG synthesis in leaves, rather than β-oxidation, function in storage of excess fatty acids, a process that has been referred to as a cellular vent^10^ or a transient buffer^19^. Lipid turnover in leaves was reported to be slow, about 4% per day^29^. With regard to the metabolic flux of TAG synthesis in the ER, acyltransferase has been suggested to have adequate capacity^10^. Therefore, Arabidopsis plants likely accumulate TAG under heat stress to remove excess 18:3 from membranes.

### Microarray transcriptome analysis revealed heat-responsive genes possibly involved in glycerolipid metabolism under heat stress

To explain the changes in lipid composition from the perspective of gene expression, microarray transcriptome analysis was performed. Arabidopsis ecotype Col-0 plants at 18 days of age were subjected to heat stress at 38 °C for one day, and then returned to 22 °C (Supplementary Fig. S1). Aerial parts of the plants were harvested at 2 time points for heat treatment (Heat08 hr, Heat24 hr) and 2 time points for recovery (Recovery08 hr, Recovery24 hr). Because the transcriptome data from normally grown plants were similar when they were harvested at the same clock time, mean values for the 22 °C control condition (Control08 hr) were calculated from the transcriptome data of both 18-day-old and 19-day-old plants harvested at the same clock time of Heat08 hr and Recovery08 hr. Similarly, the other control condition (Control24 hr) was averaged from the data among 18-day-old, 19-day-old, and 20-day-old plants harvested at the same clock time of Heat24 hr and Recovery24 hr.

#### Overview of changes in glycerolipid metabolism

**Figure 3:**
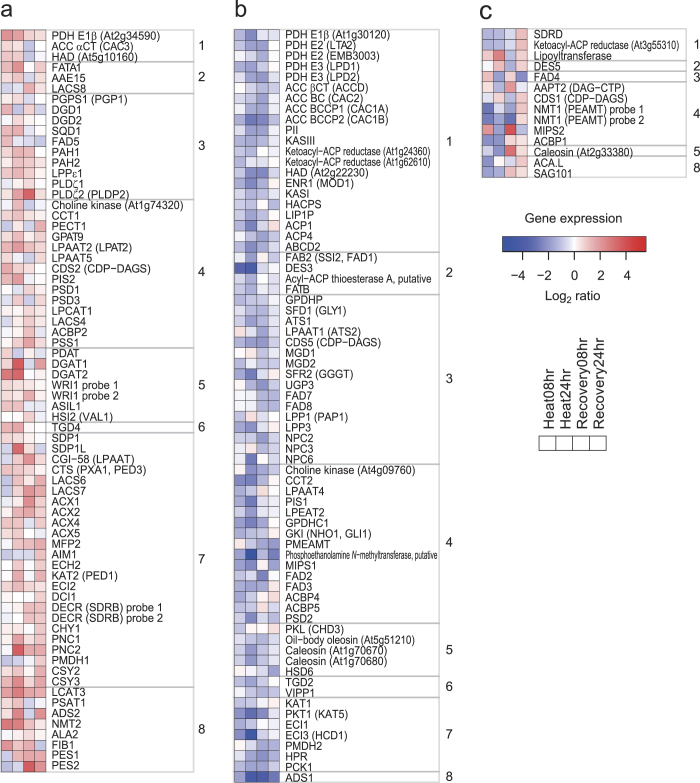
The expression of genes annotated to be involved in glycerolipid metabolism in Arabidopsis leaves under heat stress and during recovery. The expression of 233 genes (228 probe sets) selected from the literature was analysed by microarray. Four sets of fold-changes were calculated as Heat08 hr/Control08 hr, Heat24 hr/Control24 hr, Recovery08 hr/Control08 hr and Recovery24 hr/Control24 hr and are shown as a heat map from left-to-right. (**a**) Probe sets increased >1.5-fold under at least one condition and not decreased, (**b**) probe sets decreased <0.5-fold under at least one condition and not increased, (**c**) probe sets both increased >1.5-fold under at least one condition and decreased <0.5-fold under at least one condition. Genes were selected from the literature, and classified into 8 groups of metabolic pathways: (1) plastidial fatty acid synthesis; (2) fatty acid elongation, desaturation and export from plastid; (3) plastidial glycerolipid, galactolipid and sulfolipid synthesis; (4) eukaryotic phospholipid synthesis; (5) TAG synthesis; (6) lipid trafficking; (7) TAG degradation, β-oxidation, the TCA/glyoxylate cycles; (8) miscellaneous lipid-related genes, and phytyl ester synthases. Genes that changed significantly (*t*-test with Benjamini-Hochberg FDR, *p* < 0.05, N = 3, 6, or 9) among the selected genes are shown. Further information is available in Supplementary Table S2.

From the literature, 244 genes were selected as possibly being involved in glycerolipid metabolism^11^,^30^,^31^,^32^ (Supplementary Table S2). Among them, the expression levels of 233 genes were detectable as signal intensities from 228 probe sets; the genes exhibiting significantly increased (>1.5-fold) and decreased (<0.5-fold) expression were analysed. Because the number of genes with increased expression was smaller, we analysed the >1.5-fold, but not the >2-fold data. The samples at Heat08 hr, Heat24 hr, Recovery08 hr, and Recovery24 hr contained 31, 48, 38, and 38 increased probe sets, respectively (Supplementary Fig. S5). On the other hand, they also contained 41, 54, 44, and 13 decreased probe sets, respectively (Supplementary Fig. S5). Recovery24 hr contained the smallest number of genes (13) with decreased expression, suggesting that the plants quickly recovered from the stress at least at the transcriptional level. In total, there were 68 genes (70 probe sets) with increased expression under at least one condition and no decrease, 71 genes (69 probe sets) with decreased expression under at least one condition and no increase, 13 genes (13 probe sets) with both increased and decreased expression under at least one condition, and 81 genes (76 probe sets) with neither increased nor decreased expression (Fig. 3, Supplementary Table S2).

#### Stress-responsive transcription factors

The expression of transcription factor genes involved in abiotic stress in Arabidopsis was examined (Supplementary Fig. S6, Supplementary Table S3). *DREB1A*, which was reported to be involved in cold stress^33^, decreased <0.5-fold at Heat08 hr and Recovery08 hr. *DREB2A*, which was reported to be involved in drought and heat stress^34^,^35^, increased >2-fold at Heat08 hr, Heat24 hr, Recovery08 hr, and Recovery24 hr. *HSFA2*, which was reported to be involved in heat stress^36^, increased >2-fold at Heat08 hr, Heat24 hr, and Recovery08 hr. These results indicate that appropriate heat-stress responses of Arabidopsis leaves were detectable in our experimental conditions.

#### Circadian changes in glycerolipid metabolism-relate gene expression

The observation that some genes displayed different expression levels between Control08 hr and Control24 hr suggests an effect of circadian rhythm on their expression. There were 17 probe sets that increased >1.5-fold at Control24 hr (i.e., in the morning), whereas 24 sets increased >1.5-fold at Control08 hr (i.e., in the afternoon) at 22 °C (Supplementary Table S2). Among them, 40 probe sets changed under heat stress or during recovery. Therefore, increase in the expression of *DGD1* (digalactosyldiacylglycerol synthase 1), *FAD5* (fatty acid desaturase 5), *PDAT* (phospholipid: diacylglycerol acyltransferase), *FIB1* (fibrillin 1), *FAD4*, and *MIPS2* (*myo*-inositol-1-phosphate synthase 2) at Heat08 hr may reflect a heat stress-induced decrease in circadian-influenced expression of genes related to glycerolipid metabolism.

#### Prokaryotic glycerolipid pathway in chloroplasts

The expression of 21 genes, which are likely involved in *de novo* fatty acid synthesis in chloroplasts, was decreased by heat stress and/or during recovery (group 1 of Fig. 3b). *ATS1* (glycerol-3-phosphate acyltransferase 1), *LPAAT1* (1-acylglycerol-3-phosphate acyltransferase 1), and *CDS5* (CDP-diacylglycerol synthase 5) are involved in the so-called prokaryotic pathway, which refers to the plastidic biosynthesis of 16:3-containing glycolipids and PG^30^. The expression of these genes decreased at Heat08 hr, Heat24 hr, and/or Recovery08 hr (Fig. 3b). These results suggest a decrease in chloroplast glycerolipid synthesis, which is consistent with the decrease observed in 34:x glycolipids and PG under heat stress and during recovery (Fig. 2). Decrease in the expression of *MGD1* (monogalactosyldiacylglycerol synthase 1) and *MGD2* at Recovery08 hr can explain the lipidomics results, in which the total amount of MGDG decreased during recovery (Supplementary Fig. S7). MGDG and PG may be also converted to TAG and other lipids, which have been detected after wounding^27^. FAB2 (also referred to as SSI2 and FAD1) converts 18:0-ACP to 18:1-ACP in chloroplasts^37^. Its expression decreased at Heat24 hr, which may explain the increased accumulation of 18:0-containing 36:1 PC under heat stress (Fig. 2).

#### Phospholipid synthesis in the ER

**Figure 4:**
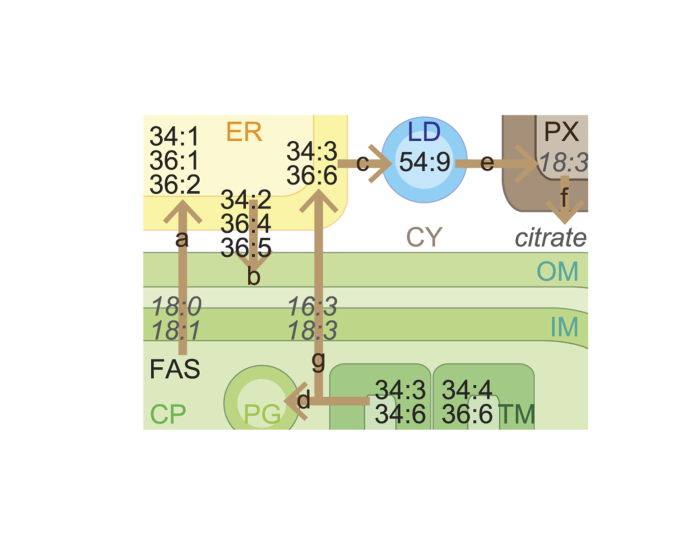
Predicted intracellular lipid trafficking. Integrated lipidome and transcriptome investigations suggest an enhanced lipid movement among organelles under heat stress in Arabidopsis leaves. Seven arrows (‘**a**’ through ‘**g**’) show metabolic pathways suggested to be induced by heat stress. FAS, fatty acid synthesis; CP, chloroplasts; TM, thylakoid membrane; PG, plastoglobules; IM, inner envelope membrane; OM, outer envelope membrane; ER, endoplasmic reticulum; LD, lipid droplets; PX, peroxisome; CY, cytosol.

FATA1 prefers to hydrolyse 18:1-ACP rather than 16:0-ACP in chloroplasts^38^. Mutations resulting in reduced *FATA1* gene expression were reported to affect fatty acid composition in Arabidopsis seeds^39^. The expression of *FATA1*, *PAH1* (phosphatidate phosphohydrolase 1), *PAH2*, *CCT1* (choline-phosphate cytidylyltransferase 1) and *LPAAT2* increased at Heat08 hr and/or Heat24 hr (Fig. 3a). These observations could explain the increase in 18:1-containing 34:1 PC, 36:1 PC, 36:2 PC, 34:1 PE, 36:2 PE, and 34:1 PI in extrachloroplasts under heat stress (Fig. 2, pathway ‘a’ of Fig. 4).

#### Eukaryotic glycolipid synthesis

PAH1 and PAH2 were reported to be involved in the transport of diacyl glycerolipids from the ER to chloroplasts under phosphorus-depleted stress^40^. *LPPε1* encodes one of the chloroplast-localised phosphatidic acid (PA) phosphatases^41^. *PLDζ2* was reported to be induced by phosphorus deficiency and be involved in phospholipid metabolism^42^. TGD4 was reported to bind to PA and be involved in the transport of glycerolipids from the ER to chloroplasts^43^,^44^. The expression of these genes increased at Heat08 hr and/or Heat24 hr (Fig. 3a). This result could explain the decrease in 18:2-containing 34:2 PC, 36:4 PC, 36:5 PC, 34:2 PE, 36:4 PE, 36:5 PE, and 34:2 PI accumulated in extrachloroplasts, as well as the increase in 18:2-containing glycolipids including 36:4 MGDG, 36:5 MGDG, 36:4 DGDG, 36:5 DGDG, 36:4 SQDG, and 36:5 SQDG, which are synthesised via the so-called eukaryotic pathway, in which the substrates of glycolipid fatty acids are derived from the ER (Fig. 2, pathway ‘b’ of Fig. 4). These data reveal drastic changes in the flow of DAG in glycolipid metabolism accompanied by changes in the expression of genes involved in phospholipid degradation and lipid transport from the ER to chloroplasts. In the case of SQDG, UDP-sulfoquinovose may be newly synthesised under heat stress because the expression of *SQD1* (UDP-sulfoquinovose synthase) increased (Fig. 3a).

#### TAG synthesis

The expression of *PAH1*, *PAH2*, *PLDζ2*, *LPAAT2*, *PDAT*, *DGAT1* (acyl-CoA: diacylglycerol acyltransferase 1), and *DGAT2* increased at Heat08 hr and/or Heat24 hr (Fig. 3a). These genes may be involved in increases in TAG synthesis in the ER via the activation of the Kennedy pathway under heat stress (Fig. 2, pathway ‘c’ of Fig. 4). *FIB1a* and/or *FIB1b*, which are recognised by the same probe set, were reported to be involved in plastoglobule formation in chloroplasts under abiotic stress^45^. Their expression increased at Heat08 hr, Heat24 hr, and Recovery08 hr, suggesting the involvement of these genes in plastoglobule formation under heat stress (Fig. 3a). The expression of *PES1*^11^ and *DGAT1*^46^, which were reported to be involved in TAG synthesis in chloroplasts, increased under heat stress and during recovery (Fig. 3a). TAG might also accumulate in chloroplasts as plastoglobules (pathway ‘d’ of Fig. 4).

#### Genes involved in lipid turnover

The expression of *SDP1* and *SDP1L*, which hydrolyse TAG^47^, increased at Heat08 hr and Heat24 hr, respectively (Fig. 3a). These results suggest that, although TAG may accumulate, TAG degradation is also enhanced under heat stress (pathway ‘e’ of Fig. 4). The expression of 21 genes, which are likely involved in β-oxidation and the tricarboxylic acid (TCA) and glyoxylate cycles, increased at Heat24 hr, Recovery08 hr, and Recovery24 hr (group 7 of Fig. 3a). These results suggest that Arabidopsis increases turnover of membrane glycerolipids under heat stress and during recovery at the transcriptional level (pathway ‘f’ of Fig. 4).

In germinating Arabidopsis, the first step of the TCA cycle is catalysed by *CSY2* and *CSY3*, which were reported to be localised in the peroxisome^48^. The authors showed that these proteins are essential for exporting citrate derived from acetyl-CoA from the peroxisome to the cytosol for fatty acid respiration. The expression of these genes increased under heat stress and during recovery (Fig. 3a). Conversely, the expression of other genes (*PMDH2*, *HPR*, and *PCK1*), which also belong to the glyoxylate cycle and have been suggested to be involved in lipid catabolism^32^, did not increase under heat stress (Fig. 3b). These results suggest that Arabidopsis produces citrate in the peroxisome to effect changes in membrane composition, rather than use lipids as energy sources under heat stress (pathway ‘f’ of Fig. 4).

## Conclusion

The lipidomics data indicate that the composition of membrane lipids in leaves was dynamic under heat stress within one day (Fig. 2). Most of these changes can be understood by a decrease in unsaturated fatty acids from chloroplast membranes. This decrease was likely derived from (1) lipid transfer between chloroplasts and the ER, and (2) decrease in chloroplast glycolipids and PG containing 18:3 and 16:3 by lipid turnover (pathways ‘a’ through ‘f’ of Fig. 4). This process was likely enhanced by increases in 18:3-containing TAG species. In most cases, microarray results suggest the regulation of glycerolipid metabolism at the transcriptional level. However, for lack of gene annotations, the process of degradation and export of chloroplastic 16:3 and 18:3 cannot be explained (pathway ‘g’ of Fig. 4). The integration of the lipidomics data and transcriptome data will be useful for elucidation of the unknown pathways in the future.

## Methods

### Lipidomic analysis

*Arabidopsis thaliana* ecotype Columbia (Col-0) and Nossen were used. Arabidopsis seeds were surface-sterilised and sown on an agar-solidified Murashige and Skoog medium containing 0.5% (*w/v*) sucrose. Plants were grown at 22 °C under a 16-h-light/8-h-dark cycle. Fourteen-day-old Col-0 and Nossen plants at about 3 h after the onset of the light phase were subjected to heat stress at 22 °C (control), 30 °C, 34 °C or 38 °C for one day under a continuous light condition (biological replicate N = 4) (Supplementary Fig. S1). For recovery experiments, 18-day-old Col-0 and 14-day-old Nossen plants were subjected to heat stress at 38 °C for one day under the 16/8 h light-dark cycle, and then returned to 22 °C and grown for one day and 2 days longer (biological replicate N = 3, 4, 8, or 12) (Supplementary Fig. S1).

Aerial parts were harvested at about 3 h after the onset of light phase. Crude lipid was extracted from the aerial parts according to the method of Okazaki *et al.*^49^. The dried chloroform extract was dissolved in 160 μL ethanol for LC-MS analysis. The conditions for LC-MS analysis were described in the same paper^49^. Lipid data were obtained from 77 distinct plant samples that included 6 environmental conditions and 2 ecotypes, in which the plant samples were prepared from 6 individual growth periods.

Levels of individual lipid species in every sample preparation were normalised to the sum of peak areas of lipid species within the same lipid class in samples grown at 38 °C for one day (1d38C). The scaled levels of the 66 lipid species, which were detected under the all conditions and ecotypes, were calculated to show overview as a heat map, where levels of individual lipid species were subtracted by the mean of values of the lipid species among the 77 samples, then divided by the standard deviations for values of the lipid species among the 77 samples as described in Vu *et al.*^27^. Spearman’s correlation coefficients were calculated among 12 variables, which were expressed as the average of each condition (22 °C control (1d22C and 1d22C+2d22C), 30 °C for one day (1d30C), 34 °C for one day (1d34C), 1d38C, 38 °C for one day then 22 °C for one day (1d38C+1d22C), and 38 °C for one day then 22 °C for 2 days (1d38C+2d22C)) for each ecotype (Col-0 (C) and Nossen (N)). Hierarchical clustering of the lipid species was conducted by the complete linkage method based on Euclidean distance (k = 10) by the software R. A heat map was constructed to depict the log_2_ of the ratio of the average of each stress condition to that of the 22 °C control. Significant differences in individual values were investigated by Welch’s *t* test (two-sided).

### Transcriptome analysis

Arabidopsis ecotype Col-0 seeds were surface-sterilised and sown on an agar-solidified medium, which was identical to that used for lipidomic analysis as described above. Plants were grown at 22 °C under a 16-h-light/8-h-dark cycle. Eighteen-day-old plants were subjected to heat stress at 38 °C for 24 h (Supplementary Fig. S1). The stress treatment was started at 3 h after the onset of the light phase. After the heat stress treatment, the plants were grown at 22 °C for 24 h.

Aerial parts were harvested at 8 h after the start of heat stress (Heat08 hr, biological replicate N = 3), 24 h after the start of heat stress (Heat24 hr, biological replicate N = 3), 8 h after the start of recovery (Recovery08 hr, biological replicate N = 3), and 24 h after the start of recovery (Recovery24 hr, biological replicate N = 3) (Supplementary Fig. S1). The samples named Control08 hr (N = 6, including 3 biological replicates of 18-day-old and 19-day-old plants) and Control24 hr (N = 9, including 3 biological replicates of 18-day-old, 19-day-old and 20-day-old plants) were harvested from plants grown under the 22 °C condition at 11 and 3 h after the onset of light phase, respectively.

Total RNA was extracted using an RNeasy Plant Mini Kit (Qiagen, Valencia, CA). The Arabidopsis Genome ATH1 DNA array (Affymetrix, Santa Clara, CA) was hybridised according to the manufacturer’s protocol. Raw data were deposited in NCBI-GEO under accession numbers GSE63128. The obtained data were summarised by the method of MAS5, and normalised as baseline to median of all samples using GeneSpring version 12 (Agilent Technologies, Palo Alto, CA).

Genes possibly involved in glycerolipid metabolism were selected from the literature (Supplementary Table S2)^11^,^30^,^31^,^32^. There were 244 genes annotated as being involved in the metabolic pathways: plastidial fatty acid synthesis; fatty acid elongation, desaturation and export from the plastid; plastidial glycerolipid, galactolipid and sulfolipid synthesis; eukaryotic phospholipid synthesis; triacylglycerol (TAG) synthesis; lipid trafficking; TAG degradation; β-oxidation; the tricarboxylic acid cycle and glyoxylate cycle (selected only the genes possibly related to fatty acid catabolism^32^); miscellaneous lipid-related (selected only the genes having gene symbols^31^); and phytyl ester synthases^11^ (Supplementary Table S2). The genes showing significant changes in expression were chosen by volcano plots of fold-changes and the moderated *t*-test with the Benjamini-Hochberg FDR method (corrected *p*-values) among the selected genes (or the whole probe sets for analysis of transcription factors) by the software GeneSpring.

## Additional Information

**How to cite this article**: Higashi, Y. *et al*. Landscape of the lipidome and transcriptome under heat stress in Arabidopsis thaliana. *Sci. Rep.* **5**, 10533; doi: 10.1038/srep10533 (2015).

## Supplementary Material

Supporting Information

Supporting Information

Supporting Information

Supporting Information
