# Supplementary material for: Landscape of the lipidome and transcriptome under heat stress in Arabidopsis thaliana
Source: Sci Rep. 2015 May 27;5:10533. doi: 10.1038/srep10533 (PMC4444972; doi:10.1038/srep10533)
Supplement: Supporting Information [file srep10533-s1.pdf]

Title

Landscape of the lipidome and transcriptome under heat stress in *Arabidopsis thaliana*

Author list and affiliations

Yasuhiro Higashi[1], Yozo Okazaki[1], Fumiyoshi Myouga[1], Kazuo Shinozaki[1], Kazuki Saito[1,2]

[1]RIKEN Center for Sustainable Resource Science, 1-7-22 Suehiro-cho, Tsurumi-ku, Yokohama, Kanagawa 230-0045, Japan, and [2]Graduate School of Pharmaceutical Sciences, Chiba University, Inohana 1-8-1, Chuo-ku, Chiba 260-8675, Japan

Supplementary information

The following materials are available in the online version of this article.

Supplementary Figure S1. Plant growth condition for lipidomic and microarray analysis.

Supplementary Figure S2. Phenotypes of heat stress-treated rosettes.

Supplementary Figure S3. Spearman's correlation coefficients of lipid species.

Supplementary Figure S4. Relative amount of TAG species.

Supplementary Figure S5. Venn diagrams of heat-responsive genes annotated to be involved in glycerolipid metabolism.

Supplementary Figure S6. The expression of known transcription factor genes involved in abiotic stress response in Arabidopsis.

Supplementary Figure S7. Changes in lipid classes.

Supplementary Table S1. Lipidomics data.

Supplementary Table S2. Microarray data of genes involved in glycerolipid metabolism including putative ones.

Supplementary Table S3. Microarray data of the whole probe sets.

The Supplementary Tables are available as Microsoft excel files.

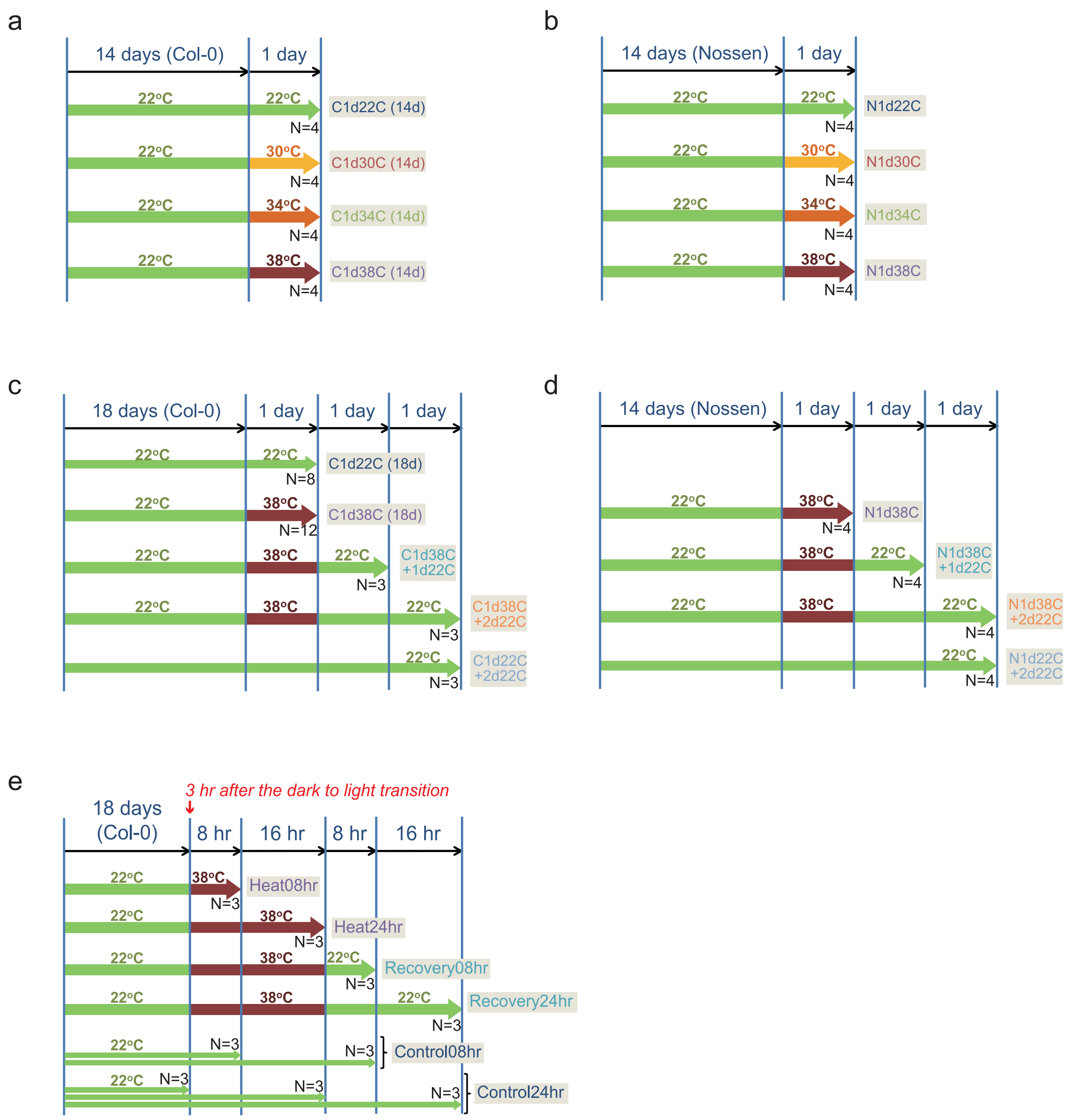

Supplementary Figure S1. Plant growth condition for lipidomic and microarray analysis. Arabidopsis ecotype Col-0 and Nossen were grown on MS media agar plates at different temperatures (22ºC, 30ºC, 34ºC, 38ºC) for a period of one day. After the 38ºC stress, Arabidopsis plants were transferred to a normal growth chamber at 22ºC and grown 1 day or 2 days longer. Total lipids were extracted from the Arabidopsis leaves. Lipidomic analysis of (a) Col-0 under heat stress, (b) Nossen under heat stress, (c) Col-0 during recovery, and (d) Nossen during recovery. (e) Microarray analysis. N = the number of biological replicates.

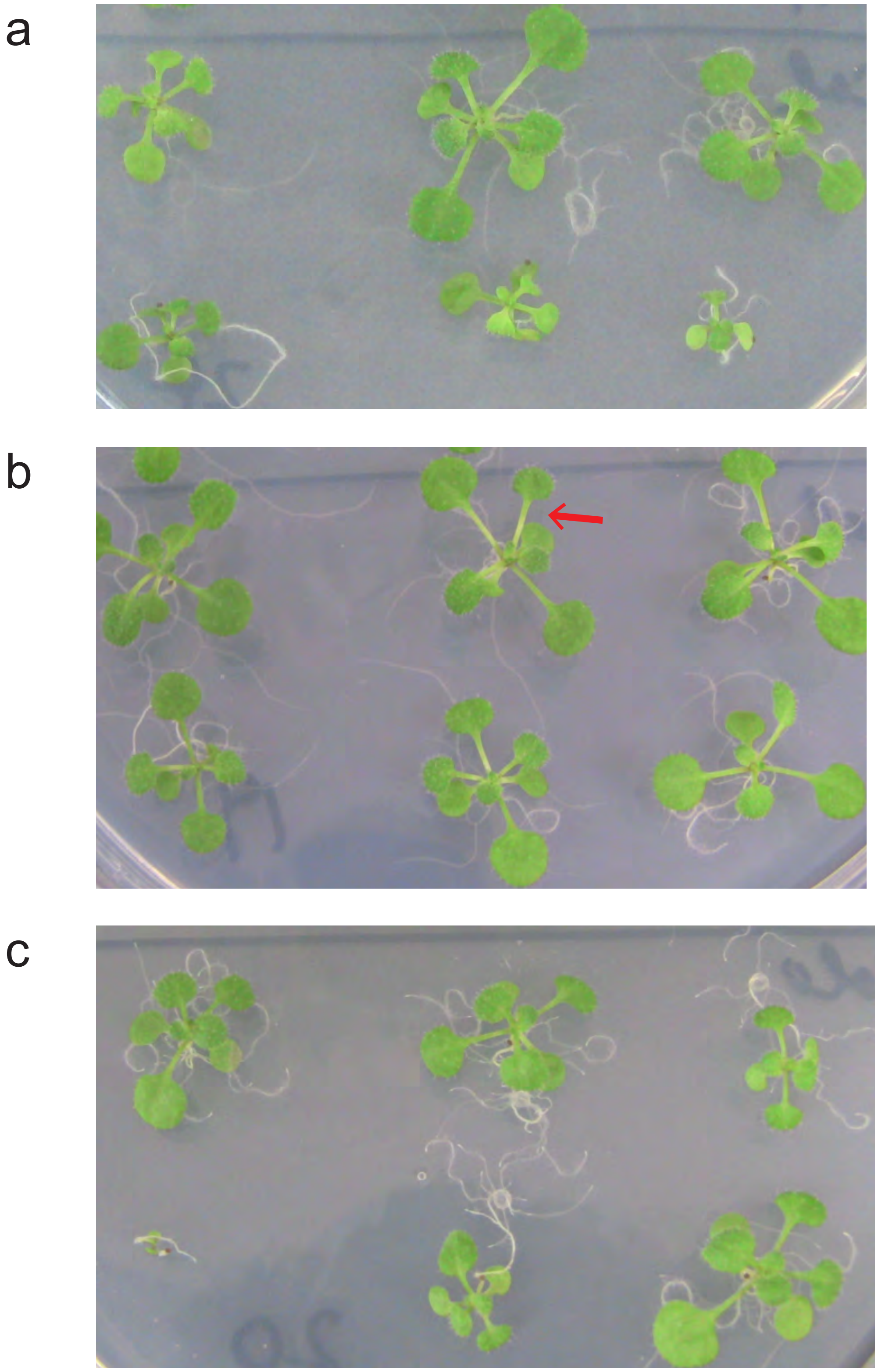

Supplementary Figure S2. Phenotypes of heat stress-treated rosettes. Arabidopsis ecotype Col-0 14-day-old plants were incubated at different temperatures: (a) 22ºC as a control, (b) 30ºC, and (c) 38ºC for a day. Red arrow shows petiole elongation.

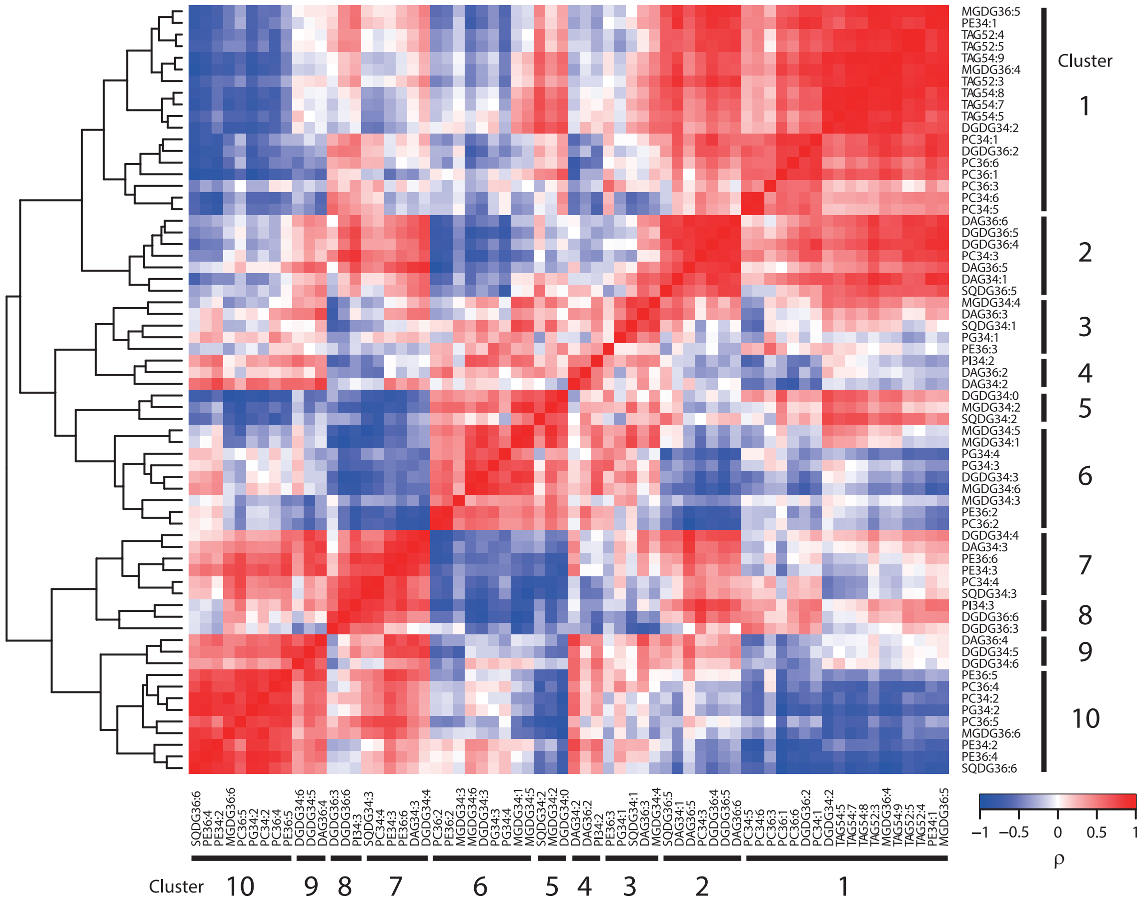

Supplementary Figure S3. Spearman's correlation coefficients of lipid species. The observed 66 lipid species were normalised to 1d38C data in each experiment. Spearman's correlation coefficients of the lipid species were calculated from averages of 2 genotypes and 6 environments (12 variables). A control was calculated as an average of both conditions: 1d22C and 1d22C+2d22C. The lipid species were clustered into 10 groups by the complete linkage method based on Euclidean distance by the software R. The colour scales show the coefficient (ρ).

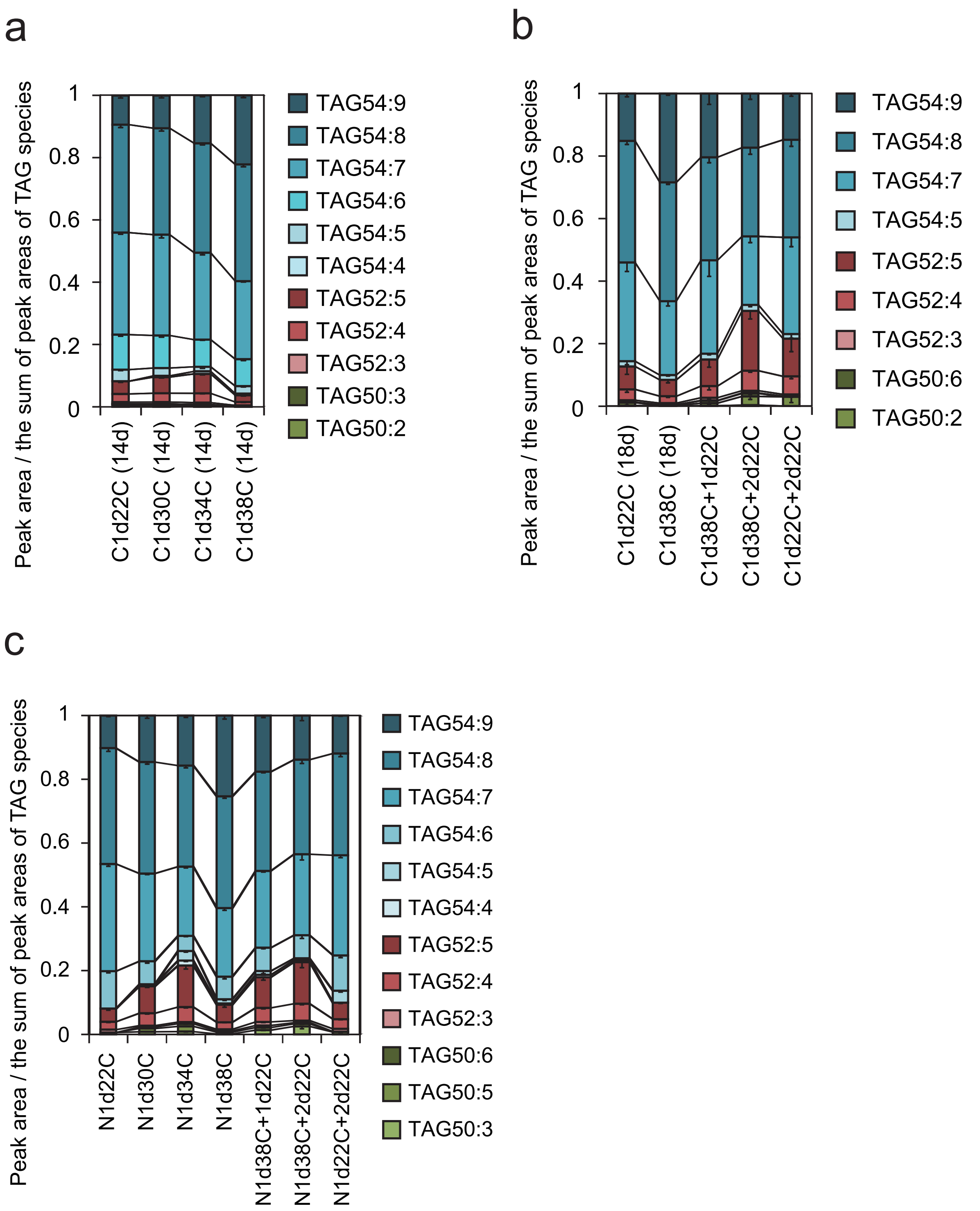

Supplementary Figure S4. Relative amount of TAG species. Relative amount of each TAG species is shown as the ratio of the sum of values from each TAG species detected under each stress condition. (a) Col-0 14-day-old (N = 4), (b) Col-0 18-21-day-old (N = 3, 8, or 12), and (c) Nossen 14-17-day-old (N = 4 or 8). Each data point represents the mean value ± SD.

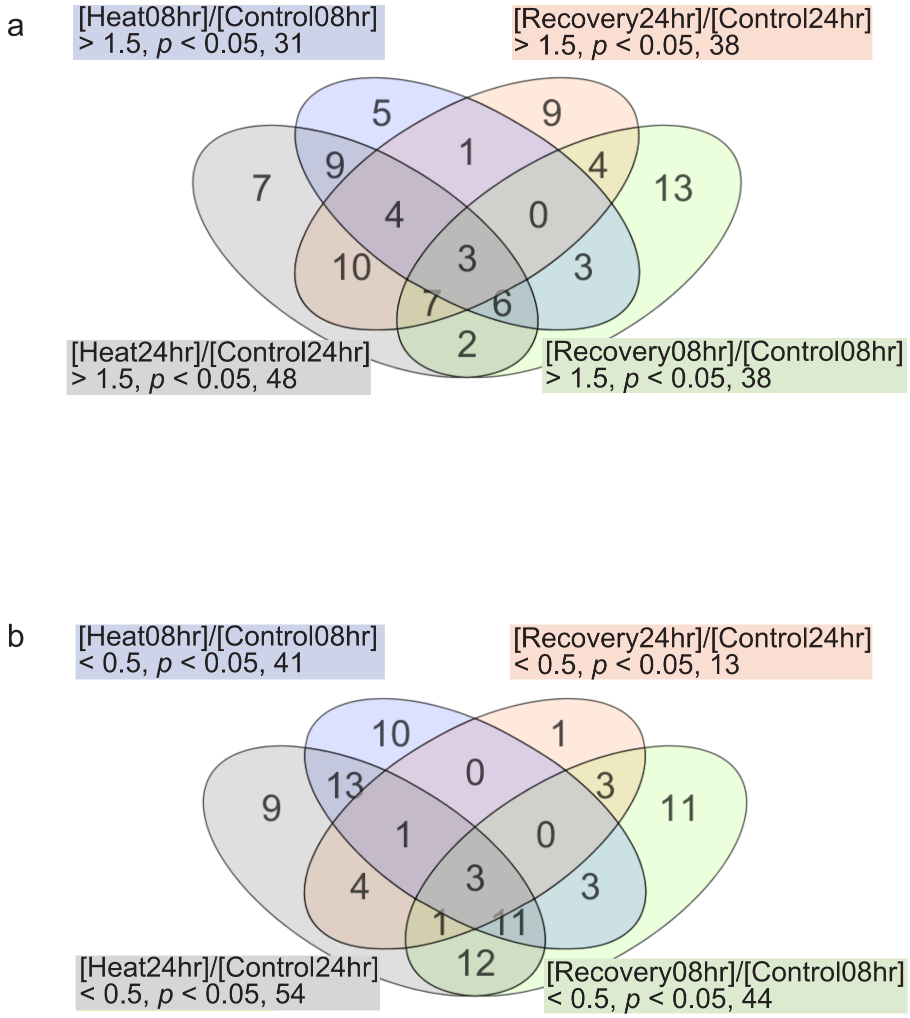

Supplementary Figure S5. Venn diagrams of heat-responsive genes annotated to be involved in glycerolipid metabolism. Microarray data were obtained from 6 conditions, Control08hr, Control24hr, Heat08hr, Heat24hr, Recovery08hr and Recovery24hr. After normalization and filtering, the expression of genes annotated to be involved in glycerolipid metabolism was analysed. Four sets of fold-changes were calculated as Heat08hr/Control08hr, Heat24hr/Control24hr, Recovery08hr/Control08hr and Recovery24hr/Control24hr. Genes with significant changes in expression (*t*-test and Benjamini-Hochberg FDR, $p < 0.05$, N = 3, 6, or 9) among the analysed genes were selected. Venn diagrams show overlapped probe sets of (a) genes upregulated > 1.5-fold, and (b) genes downregulated < 0.5-fold.

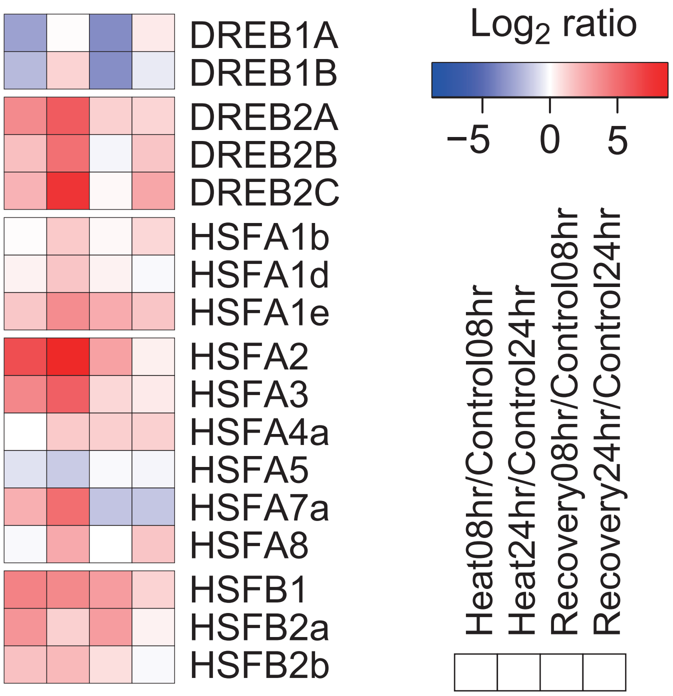

Supplementary Figure S6. The expression of known transcription factor genes involved in abiotic stress response in Arabidopsis. Genes with significantly increased > 2.0-fold (red) and decreased < 0.5-fold (blue) expression are shown (*t*-test and Benjamini-Hochberg FDR, $p < 0.05$, N = 3, 6, or 9, among the whole probe sets). Further information is available in Supplementary Table S3.

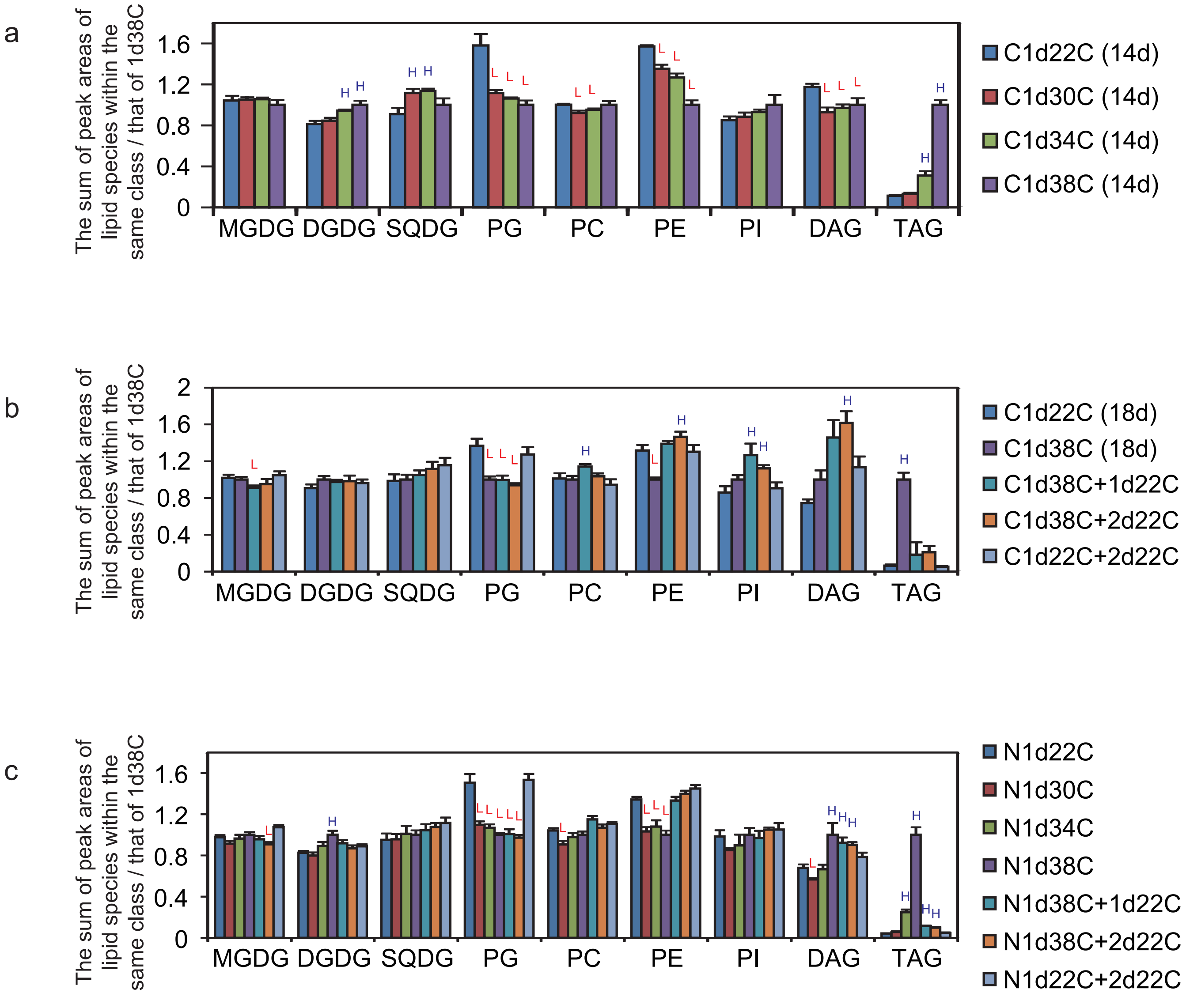

Supplementary Figure S7. Changes in lipid classes. The total amount of each lipid class is shown as the sum of peak areas of lipid species within the same class divided by that of 1d38C (purple) from each experiment. L, H: Welch's *t*-test: (a) Col-0 14-day-old, $p < 0.01$ from C1d22C (14d), N = 4; (b) Col-0 18-21-day-old, $p < 0.05$ from both C1d22C (18d) and C1d22C+2d22C, N = 3, 8, or 12; (c) Nossen 14-17-day-old, $p < 0.01$ from both N1d22C and N1d22C+2d22C, N = 4 or 8. Each data point represents the mean value ± SD.
